# Turnover prevention: The direct and indirect association between organizational job stressors, negative emotions and professional commitment in novice nurses

**DOI:** 10.1111/jan.14281

**Published:** 2019-12-17

**Authors:** Yvonne ten Hoeve, Jasperina Brouwer, Saskia Kunnen

**Affiliations:** ^1^ Health Sciences – Nursing Research University of Groningen/University Medical Center Groningen Groningen the Netherlands; ^2^ Faculty Behavioural and Social Sciences Department of Educational Sciences University of Groningen Groningen the Netherlands

**Keywords:** emotions, job stressors, novice nurses, professional commitment, support, turnover, work experiences

## Abstract

**Aims:**

Getting insight in the most crucial organizational job stressors for novice nurses' professional commitment and whether the job stressors are mediated through negative emotions.

**Design:**

The study used an observational cohort design.

**Methods:**

Organizational job stressors were derived from 580 diary entries by 18 novice nurses combined with measures on emotions and commitment. The diaries were collected from September 2013–September 2014.

**Results:**

Path modelling revealed that lack of support from colleagues, negative experiences with patients and confrontations with existential events were most strongly negatively related to professional commitment through negative emotions. Other indirectly and negatively related organizational job stressors to commitment were complexity of care, lack of control and work‐life imbalance; only conflicting job demands, and lack of control related to professional commitment directly.

**Conclusion(s):**

To enhance professional commitment, it is important to reduce negative emotions in novice nurses by collegial support in dealing with negative experiences with patients, complexity of care and existential events and to prevent lack of control and an imbalance between private life and work. Nurse supervisors and managers can encourage nurses to share negative patient experiences, issues related to complexity of care and existential events.

**Impact:**

Considering the worldwide nursing shortage and early turnover, more understanding is needed about how negative emotions mediate the relationship between organizational negative job stressors and professional commitment and the relative impact of organizational job stressors to professional commitment. The study stresses the importance of a supportive role of supervisors and nurse managers to improve the work environment and hence increase novice nurses' commitment and retention.

## Abstract (zh)

**目的:**

了解新手护士专业承诺中最为突出的组织工作压力源,以及工作压力源是否通过负面情绪进行调节。

**设计:**

本项研究采用观察性定群设计。

**方法:**

根据18名新手护士的580篇日记,结合情绪和承诺衡量结果,调查组织工作压力源。这些日记的收集工作于2013年9月至2014年9月进行。

**结果:**

路径模型显示,缺乏同事的支持,患者的消极经历以及与生存事件的冲突通过负面情绪与专业承诺之间呈显著负相关。对于承诺,其他间接负相关的组织工作压力源包括护理复杂度、控制不足以及工作生活失衡,仅有相互冲突的工作需求,缺乏与专业承诺直接相关的控制。

**结论:**

为加强专业承诺,在处理患者消极经历、护理复杂度和生存事件,防止出现缺乏控制、个人生活与工作失衡情况时,应通过提供同事支持来减少新手护士的负面情绪。护士长和管理者可鼓励护士分享消极的病人经历以及护理复杂度和生存事件相关问题。

**影响:**

考虑到全球范围内护理人员短缺和过早离职的局面,亟需进一步了解负面情绪如何对组织消极工作压力源与专业承诺的关系进行调节,以及组织工作压力源对专业承诺的相对影响。本项研究突出强调了护士长和护士管理者提供支持的重要性,以改善工作环境,从而坚定新手护士的承诺,增强其留职意愿。

## 1 INTRODUCTION

For decades, employee turnover has been one of the main challenges of managers because of the costs involved in recruiting and training new employees (Lee, Gerhart, Weller, & Trevor, 2008). In healthcare organizations, total turnover costs are to a large extent caused by nurse turnover rates because of the considerable size of the workforce (Waldman, Kelly, Arora, & Smith, 2010). Both costs and, importantly, gaps in the supply of care and patient outcomes are a problem. Needleman et al. (2011) showed that the risk of mortality was 6% higher on understaffed units compared with fully staffed units. Similarly, other studies have demonstrated that more nurses at the bedside can improve quality of care and reduce infections and patient mortality (Aiken, Clarke, Sloane, Sochalski, & Silber, 2002; Blegen, Goode, Spetz, Vaughn, & Park, 2011).

One of the risk factors for turnover is a low professional commitment (Li, Lee, Mitchel, Hom, & Griffeth, 2016). Professional commitment, in turn, is linked to job demands—resources and emotional exhaustion (Jourdain & Chênevert, 2010). Negative emotions, such as job dissatisfaction, might reduce professional commitment and increase the risk of turnover, whereas positive work environments might decrease turnover rates (Suliman & Aljezawi, 2018). Organizational job stressors are related to the organizational context, such as high workload, complexity of care and patient suffering (Andela, Truchot, & Van der Doef, 2015) and to inter‐personal factors, such as work‐life interference and nurses' relationships with other healthcare professionals (Liu et al., 2018; Schaefer, Zoboli, & Vieira, 2016). With regard to the current alarming shortage of nurses worldwide, it is important to investigate the relationship between professional commitment in (novice) nurses, negative emotions and organizational job stressors and, even more importantly, to identify the most crucial job stressors in terms of association with negative emotions and hence with job commitment.

### 1.1 Background

#### 1.1.1 Stressors related to workplace

Although novice nurses start working with a passion for the profession because of their motivation to help other people (Glearean, Hupli, Talman, & Haavisto, 2017; Usher et al., 2013), experiences in the first year may have a significant impact on their future career plans and professional commitment (Brown, Hochstetler, Rode, Abraham, & Gillum, 2018). Starting in clinical practice, nurses are confronted with many stressful and dissatisfying situations (Laschinger, Leiter, Day, & Gilin, 2009; Liang, Lin, & Wu, 2018) due to a heavy workload, patients with complex care demands (comorbidity) and conflicting job demands (cognitively or physically overburdened). Perceptions of increased workload and a high number of responsibilities may lead to physical and mental exhaustion and a sense of losing control over patient care (De Almeida Vicente, Shadvar, Lepage, & Rennick, 2016). Control can be seen as “the professional capability of employees to make important decisions about their work, as well as their ability to gain access to resources necessary to do their job effectively” (Boamah & Laschinger, 2016, p. E165). Previous studies have also shown that a lack of control can be seen as a major determinant of high levels of occupational stress in nurses (Gelsema, van der Doef, Maes, & Akerboom, 2005; McGrath, Reid, & Boore, 2003; Zangaro & Soeken, 2007). Lack of control can be the consequence of conflicting job demands that are either physical (being responsible for too many patients) or cognitive (the feeling or perception of incompetence). These conflicting demands can result in dissatisfaction, stress and exhaustion (Duchscher & Cowin, 2006; Flinkman & Salantera, 2015; Hussein et al., 2016). The literature has shown that conflicting demands also have an impact on nurses' perceived balance between work and personal life (Burke, 2002; Van der Heijden, Demerouti, Bakker, & Hasselhorn, 2008). Work‐life imbalance, where the demands in the domain of work and the demands of personal life are not compatible, is an important predictor of job dissatisfaction, turnover intentions and decreasing nurses' commitment to the profession (Grzywacz, Frone, Brewer, & Kovner, 2006; Kovner, Brewer, Wu, Cheng, & Suzuki, 2006).

#### 1.1.2 Stressors related to patients

Novice nurses not only experience a high workload and complex care demands but also encounter existential events, such as confrontations with severely ill and dying patients. Such emotionally charged experiences are often associated with nurses' lower well‐being and higher levels of burnout and turnover intentions (Karimi, Leggat, Donohue, Farrell, & Couper, 2014; McDonald, Jackson, Wilkes, & Vickers, 2016). Additionally, negative experiences with patients behaviour, such as verbal aggression, violence and sexual harassment, are associated with novice nurses' job satisfaction, burnout and intention to leave (Chang & Cho, 2016; Roche, Diers, Duffield, & Catling‐Paull, 2010; Viotti, Gilardi, Guglielmetti, & Converso, 2015).

#### 1.1.3 Stressors related to professional relationships

An important strategy that novice nurses use to deal with stressful situations is to share their experiences with colleagues and supervisors. Practical and emotional support from the work environment seem indispensable for guiding novice nurses in their first year of practice and keeping them motivated to remain in the profession (ten Hoeve, Kunnen, Brouwer, & Roodbol, 2018). These authors also explored direct relationships of contextual, relational, cognitive factors on, respectively, nurses' positive emotions, negative emotions and commitment (ten Hoeve, Brouwer, Roodbol, and Kunnen (2018). Negative factors, such as lack of support, were in particular negatively related to commitment. These findings call for more understanding of how professional commitment decreases and whether this is mediated through negative emotions. Furthermore, to explore which job stressors are the most crucial for professional commitment.

#### 1.1.4 The association between stressors and professional commitment

Our theoretical framework consists of two parts. The basis is the developmental process model of Bosma and Kunnen (2001). This theory elaborates how negative emotions mediate between events and commitment. It is a general theory about the mechanisms, and it does not focus on which factors may play a role. To apply these fundamental developmental mechanisms to a specific domain, we need a second theoretical model that elaborates which specific factors in that domain are expected to play a role. The model of Bosma and Kunnen states that commitment develops in interaction. Interactions that confirm existing commitments strengthen them; interactions that challenge them (in this paper we refer to them as negative interactions) result in emotional conflict and over time, this weakens the commitment. This general model can be applied to different identity domains. To specify the factors that are relevant in the domain of professional identity, we used the emotion‐centred model of the process of occupational stress of Spector and Goh (2001). This model fits in with the Bosma and Kunnen model because it also sees a central role for emotions in (professional) identity processes. Spector and Goh (2001) elaborate what type of interactions are relevant in the domain of professional identity. In their model, they proposed that environmental job stressors and perceived job stressors elicit negative emotions and behavioural, physical and psychological strains. We model professional commitment as how different organizational job stressors are related to professional commitment through perceived negative emotions.

**Figure 1:**
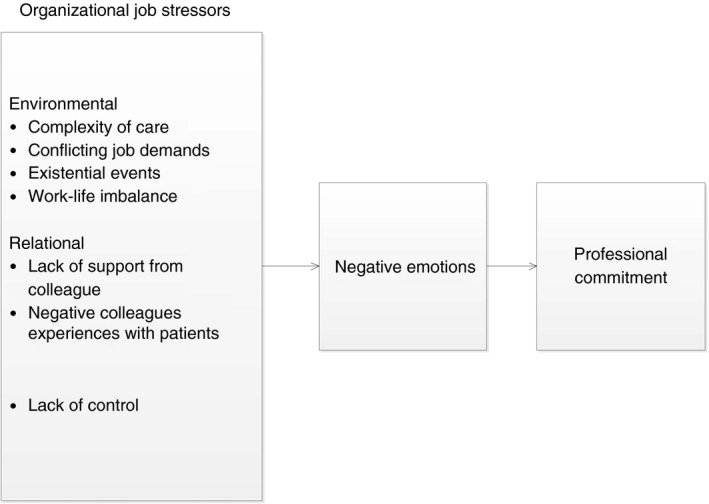
Hypothesized direct and indirect associations between organizational job stressors, negative emotions and commitment

To understand the relationship between organizational job stressors and professional commitment, it is necessary to investigate direct and indirect paths as proposed in the conceptual model (Figure 1). This study tests a theoretical model of the relationship between organizational job stressors and negative emotions and professional commitment among novice nurses. Different models such as the original Job Demands‐Resources Model (JD‐R model; Demerouti, Bakker, Nachreiner, & Schaufeli, 2001) focus on the impact of job stressors and resources on burnout and also several studies have linked job stressors to turnover intentions and burnout (see for a recent review Stevanin, Palese, Bressan, Vehviläinen‐Julkunen, & Kvist, 2018) and discussed potential turnover factors (Currie & Carr Hill 2012). Little is known, however, about the type of stressors that especially elicit negative emotions and about the mechanisms behind their effect. The current study contributes to the turnover and human resource literature because we investigated (the phenomenon of) how different job stressors may relatively affect young nurses' commitment directly and whether this is mediated by negative emotions. Some job stressors might have a stronger relationship than other job stressors with negative emotions and professional commitment. Therefore, we are also interested in the relative impact of job stressors on professional commitment. Professional commitment is an important predictor for turnover (Hayes et al., 2012), and therefore, we modelled professional commitment that might precede turnover. This knowledge is particularly important because of the need to reduce the high turnover among nurses and the shortage of nurses worldwide. The findings might be used by managers in health care for the development of strategies that prevent nurses from an early turnover.

## 2 THE STUDY

### 2.1 Aim

The aim is twofold. First, to investigate the direct and indirect relationships between organizational job stressors and novice nurses' negative emotions and professional commitment. Second, to investigate the relative impact of job stressors on professional commitment. Research questions were as follows: (a) How organizational job stressors relate to professional commitment and whether this is mediated by negative emotions?; and (b) Which organizational job stressors are most crucial for negative emotions and (hence) for professional commitment?

### 2.2 Design

This study applied an observational cohort design to examine relationships between job stressors, negative emotions and professional commitment in a convenience sample of novice registered nurses. The data that were used to answer our research questions came from a large data set with 580 diary entries from 18 novice nurses. For this study, we assessed study variables with a combination of quantitative and qualitative data collection methods. Here, we do not address changes over time, but to what extent the conceptual model fits the underlying longitudinal data.

### 2.3 Sample and setting

Participants were recruited in cooperation with the head of nursing at all wards in a University Medical Center in the Netherlands. The inclusion criteria were a Bachelor's degree in nursing, aged under 30 and with no more than 1 year's work experience (see also ten Hoeve, Brouwer, et al., 2018). A convenience sample of 18 novice nurses filled out 580 weekly records in total. All of them were female with a mean age of 23 years old (*SD* 1.43). Seven nurses followed a preliminary dual training, and eleven nurses were trained on a fulltime basis; ten nurses had clinical experience as a graduated nurse between 1 and 12 month, and eight nurses had no clinical experience; ten nurses worked as float pool nurses, whereas eight nurses worked as a staff nurse. At the end of the study, the nurses received a gift voucher by way of thanks for their participation.

### 2.4 Study variables

All study variables were assessed using a semi‐structured diary maintained by study participants. Every week participants described in their own words a positive or negative personal or work‐related experience. In our previous study (ten Hoeve, Brouwer, et al., 2018), we found that negative work experiences reduced novice nurses' commitment with their profession more than positive experiences increased commitment. For this reason, in the present study we focus only on negative experiences and the indicators of organizational job stressors.

#### 2.4.1 Organizational job stressors

A robust conceptualization of workplace stress was given by McEwen (2000) who described it as “an event or events that are interpreted as threatening to an individual and which elicit physiological and behavioural responses” (p. 173). This definition resembles the (negative) interactions in the model of Bosma and Kunnen (2001). In line with Spector and Goh (2001), we define job stressors as an interaction where negative emotions are induced. In organizational job stressors, we distinguish perceived environmental job stressors (complexity of care, conflicting job demands, existential events and work‐life imbalance) and perceived relational job stressors (lack of support from colleagues and negative experiences with patients). Rather than controlling for the variable control as in the emotion‐centred model of occupational stress of Spector and Goh (2001), we consider lack of control also as a perceived job stressor. Conducting content analysis (Miles, Huberman, & Saldaña, 2014), job stressors were coded and scored as “1” when the experience of the negative organizational job stressor was present in the described nurses' experiences and “0” when the experience was not present. For example, a negative stressor refers to a negative experience with a patient, such as a conflict.

#### 2.4.2 Emotions

Independent of the organizational job stressors (derived from the described experiences), positive and negative emotions were measured with two questions, respectively: (a) Have you felt positive emotions with regard to this experience? and (b) Have you felt negative emotions with regard to this experience? The questions were answered on a scale from 1 (not at all) to 6 (very much). In this study, we only used the scores on negative emotions.

#### 2.4.3 Professional commitment

The conceptualization of professional commitment was extensively described by Allen and Meyer (Allen & Meyer, 1990; Meyer & Allen, 1991); Meyer, Allen, & Smith, 1993). They proposed a three‐component model of organizational commitment as a psychological state. The components reflect: (a) a desire (affective commitment); (b) a need (continuance commitment); and (c) an obligation (normative commitment). Affective commitment reflects a sense of belonging, a desire to maintain membership in the profession and is most related to work experiences. In our study, professional commitment can be defined as affective commitment, as it was also related to work experiences and commitment to the profession, not to a specific job in nursing. Commitment was measured with a three‐item scale based on the Repeated Exploration and Commitment Scale in the domain of Education (RECS‐E; Van der Gaag & Kunnen, 2013). This scale is based on the process model of commitment development of Bosma and Kunnen (2001) and consists of a selection of the questions from the Identity interview GIDS (Bosma, Kunnen & van der Gaag, 2015). The three items of the scale reflect the construct professional commitment, which entails content validity of the scale (see Drenth & Sijtsma, 2006). The three items were as follows: (a) Do you stand by your choice for this particular profession? (b) Do you think that you meet the expectations of your profession? and (c) Do you feel confident in your profession? The nurses answered the questions on a Likert scale from 1 (“not at all”) to 6 (“very much”).

### 2.5 Data collection

Electronic diary and questionnaire data were collected from September 2013 to September 2014, using the Qualtrics online survey software. All nurses started at the same time and every week one of the researchers send the nurses a link to an electronic questionnaire. They were asked to describe a personal or work‐related experience from the previous week. With the following questions in mind: *“Please describe a personal or work‐related experience from the past week that really was important to you. What was the experience? In what situation? How did you reflect on this experience and how did it affect your work?*” Subsequently, they answered some quantitative questions about their emotional state, that is, whether they felt positive or negative emotions about the described experiences and their level of commitment to the profession.

### 2.6 Reliability

The factor analysis of the three commitment items revealed the following three factor loadings, respectively, 0.84, 0.93 and 0.87. The Cronbach's alpha of 0.85 indicating a good internal consistency.

### 2.7 Ethical considerations

The study was approved by the ethical committee of the Psychology Department of the University (ppo‐013–004) and all participants signed a consent form.

### 2.8 Data analysis

The nurses (*n* = 18) filled out a total of 580 experience reports (range 19–50 per participant, mean 35). Two nurses completed less than 20 reports, 13 nurses between 21 and 40 and 3 nurses completed between 41 and 50 reports. The 580 weekly reports were inductively explored using content analysis (See ten Hoeve, Brouwer, et al., 2018). The qualitative codes were quantified in dichotomized values of zeros and ones and were used as raw scores in the model together with the commitment and emotion measures of the survey. A code was labelled as one when it appeared in the weekly report and, hence, was perceived as a job stressor by the nurse in question. When it was not mentioned in the weekly report, we labelled the code as zero. The underlying data of the mode are based on these longitudinal quantitative measures in the diaries.

For this current study, descriptive statistics were conducted in SPSS 25. To gain insight into the direct and indirect paths of organizational job stressors on commitment over time, we performed a path analysis in MPlus version 7.11 (Muthén & Muthén, 1998–2013). The data set had a multilevel structure with two levels: weekly diary entries at level 1 and in nurses at level 2. Since our main interest was the quantitative relationships between these factors derived from the coded data entries, we controlled for dependency among diary entries by using the COMPLEX option, which adjusts the standard errors. Maximum likelihood (ML) estimation with robust standard errors (MLR) also deals with multivariate non‐normal and missing data. The intra‐class correlation of 0.43 for professional commitment indicated that 43% of the variance was at the nurse level compared with the total variance. For the indirect paths, we calculated bias‐corrected bootstrapped confidence intervals (Shrout & Bolger, 2002). As shown in Figure 1, we tested the conceptual model of organizational job stressors and commitment. The following indices were considered as a good model fit: a non‐significant chi‐squared test, RMSEA values less than 0.06, SRMR at 0.08 or below and CFI close to or greater than 0.95 (Hu & Bentler, 1999; Kline, 2011). It should be noted that the chi‐squared test is sensitive to sample size and can become non‐significant when the observations are large (Hu & Bentler, 1999).

## 3 RESULTS

Table 1 shows the means and the standard deviation of the professional commitment and negative emotions in each nurse. Bearing in mind the range on the answering categories on professional commitment and negative emotions from 1 to 6, the nurses score on average relatively high on professional commitment and low on negative emotions.

**Table 1:** Means and standard deviations of professional commitment and negative emotions within each nurse

Nurses (*n*; level 2)	Diary entries (*n*; level 1)	Professional commitment		Negative emotions	
		Mean	*SD*	Mean	*SD*
1	33	4.42	0.89	3.24	1.84
2	40	3.58	0.85	2.85	1.81
3	23	3.71	0.56	3.00	1.71
4	26	4.76	0.53	2.50	1.58
5	39	4.60	0.40	2.82	1.10
6	21	5.24	0.58	3.19	1.75
7	37	4.95	0.50	2.38	1.86
8	37	4.45	0.81	2.97	1.59
9	42	5.00	0.13	3.40	0.80
10	43	5.06	1.67	3.42	1.56
11	34	4.19	0.85	2.91	1.31
12	50	3.94	0.35	3.18	1.08
13	19	5.63	0.61	2.63	1.98
14	27	4.91	0.58	2.37	1.21
15	19	4.54	0.34	3.05	1.72
16	27	4.85	0.38	3.70	1.92
17	37	4.44	0.58	3.70	1.43
18	26	4.97	0.23	2.58	1.58

**Figure 2:**
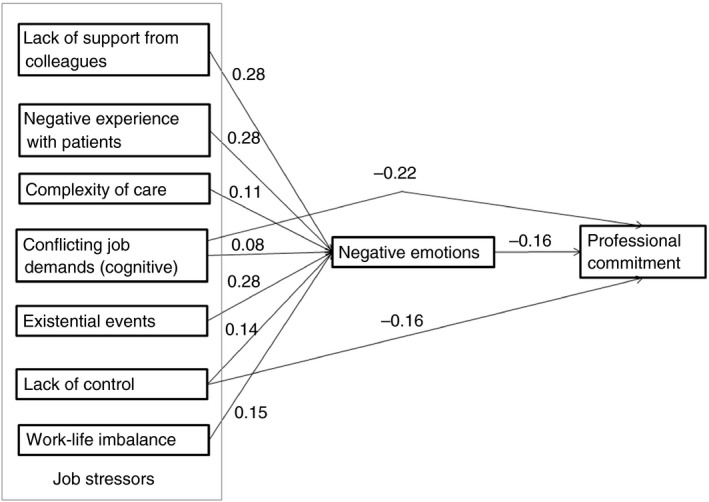
Model of organizational job stressors on negative emotions and professional commitment

To test the theoretical and conceptual model (Figure 1) featuring the expected direct and indirect relationships of stressors on professional commitment in novice nurses, we conducted path analyses. Figure 2 shows the model with standardized estimates for the direct and indirect paths. The full model with direct and indirect paths is identified with zero degrees of freedom. As recommended in Byrne (2012), we trimmed the model for methodology reasons by deleting non‐significant paths in two steps to find a parsimonious model that fits the data well. First, we deleted the paths of negative experiences with patients and existential events on commitment and second, the paths of complexity of care and work‐life imbalance on commitment. By following this approach, we obtained a final and parsimonious model. This model fit the data well: *χ* ^2^(5) = 14.68, *p* = .01, CFI = 0.960, RMSEA = 0.058 [0.03; 0.09] and SRMR = 0.022. The chi‐squared value was significant, which might be the result of 580 observations. The unstandardized beta‐estimates and standard errors of this final model are shown in the Appendix (Table 1). The model suggested several indirect paths and we found indirect relationships for all the variables except for conflicting job demands (b* = −0.01, *p* = .09), as illustrated in Figure 2. Conflicting job demands was directly and negatively related to professional commitment (b* = 0.22, *p* < .001) and directly positively related to negative emotions (b* = 0.08, *p* = .02). We found significant indirect negative paths on professional commitment through negative emotions for lack of support from colleagues (b* = −0.04, 95% CI [−0.07; −0.02]), negative experiences with patients (b* = −0.04, 95% CI [−0.07; −0.02]), complexity of care (b* = −0.02, 95% CI [−0.03; −0.001]), existential events (b* = −0.04, 95% CI [−0.07; −0.02]), lack of control (b* = −0.02, 95% CI [−0.04; −0.01]) and work‐life balance (b* = −0.02, 95% CI [−0.04; −0.01]). Existential experiences, negative relationships with colleagues and negative experiences with patients were the strongest related to negative emotions, followed by a lack of balance between personal and work life and complexity of care. Subsequently, negative emotions were negatively related to commitment. Confrontations with conflicting job demands, for example, when nurses were cognitively overburdened and lacked control over their work, were directly and negatively related to commitment and to some extent indirectly related via negative emotions.

## 4 DISCUSSION

The first research question was how organizational job stressors relate to professional commitment and whether this is mediated by negative emotions. The organizational job stressors lack of support from colleagues, negative experiences with patients, complexity of care, existential events, lack of control and work‐life balance were indirectly related to professional commitment through negative emotions. Only conflicting job demands and lack of control were directly and negatively linked to professional commitment. These findings are supported by Lanz and Bruk‐Lee (2017) in their survey among 97 nurses working at a variety of medical units in the United States. They found that job‐related negative emotions mediated the relationship between job stressors and nurses' turnover intentions.

The second research question was which organizational job stressors are most crucial for negative emotions and (hence) for professional commitment. This implies that we investigated the relative contribution of organizational job stressors to novice nurses' negative emotions and thus to professional commitment. The results showed that lack of support from colleagues, negative experiences with patients and existential events (severe illness and death of patients) are the strongest indirectly related to novice nurses' professional commitment through negative emotions. Conflicting job demands were more strongly directly and negatively related to professional commitment than lack of control. We are not aware of any research that explores which stressors are most crucial for professional commitment through negative emotions and by combining them in one model. The finding that negative job stressors increase negative emotions is in line with several studies, who investigated the job stressors separately rather than in one model. Viotti et al. (2015) suggest that negative experiences with patients are crucial for novice nurses' emotional state. Verbal aggression, intimidation, disrespectful behaviour and even sexual harassment can be seen as risk factors for emotional exhaustion and even burnout (Viotti et al., 2015). Lack of support for novice nurses in a demanding healthcare environment may lead to emotional distress (Chang, Chu, Liao, Chang, & Teng, 2018; Fleury, Grenier, Bamvita, & Farand, 2018) and job dissatisfaction (Adriaenssens, de Gucht, van der Doef, & Maes, 2011). Bacon (2017) showed that unexpected patient death as an existential event has a major impact on feelings of emotional distress, shock and failure in the nursing role. Zheng, Lee, and Bloomer (2018) demonstrated in their systematic review and qualitative meta‐synthesis that the sudden death of patients was experienced by novice nurses as a salient event. Based on the literature, it seems that nurses just entering the profession are emotionally ill prepared to cope with the emotional demands with which they are confronted. Given their age and experience, it is to be expected that novice nurses will be severely affected by these events, which is why support from the environment and mentoring might be helpful (Bacon, 2017; Erickson & Grove, 2007).

Overall, this study showed that organizational job stressors are mostly indirectly related to novice nurses' professional commitment through negative emotions. Lack of support from colleagues, negative experiences with patients and existential events were the most crucial job stressors for negative emotions and hence reducing professional commitment and conflicting job demands reduces professional commitment directly.

### 4.1 Implications for practice and policy

Supervisors and managers play a crucial role in supporting nurses and creating a positive work environment that may prevent turnover (see for a review Twigg & McCullough, 2014). It is essential that novice nurses are stimulated to express their negative emotions because this helps them to deal with complex and existentially challenging workplace situations. Therefore, novice nurses should be supported by colleagues at the beginning of their working careers. They should experience a welcoming workplace with the opportunity for development (Doyle et al., 2017). Managers can play a supportive role as well by encouraging collaboration and support among nurses (Twigg & McCullough, 2014) and by being sensitive to the emotions of nurses. Literature shows that managers have an important task in monitoring the affective states of their employees and being sensitive for the balance between job stressors and the capabilities of the nurses. The outcomes of the systematic review by Halter et al. (2017) on interventions to reduce nurse turnover showed that “transformative” nurse manager leadership styles had a positive impact on the decrease of turnover or the increase of retention. Kahn, Quinn Griffin, and Fitzpatrick (2018) found that nurse managers' transformational leadership was correlated to nurses' structural empowerment, which in turn was associated with nurse satisfaction, retention and organizational commitment. As Spector and Goh (2001) suggested, most job stressors cannot be simply removed. Therefore, emotional support and training or coaching in how to deal with pressure and with the complexity of tasks would be helpful. This support may overcome negative emotions and increase professional commitment. Also, mentoring for novice nurses is found to be helpful, for example, informal supervision on a structural basis and peer intervention. In their systematic review, Jokelainen, Turunen, Tossavainen, Jamookeeah, and Coco (2011) showed that mentoring creates a supportive learning environment and encourages professional development among nurses. To address disrespectful behaviour from patients, there is a strong need for empowerment among novice nurses. If they can address such behaviour adequately, this may enhance job satisfaction and commitment (Cicolini, Comparcini, & Simonetti, 2014). One opportunity would be to offer novice nurses practical courses in these domains and to pay more attention to nurse empowerment during education. It is important to find long‐term solutions; otherwise, we run the risk of creating a vicious circle where we recruit nursing students without the ability to supervise them because of staff shortages and consequently lose novice nurses because of early turnover.

### 4.2 Strengths and limitations

By aggregating the diary entries of each individual but taking into account that the data are nested, the statistical advantage and strength of the study are that the reliability of the data with more measures for each individual is higher than with one measurement for each individual. Despite the strength of the study given the number of diary entries that were collected and the mixed‐method approach, we note two important limitations. First, the transferability of the results is limited since the research was conducted among eighteen novices in only one hospital. We recommend replicating the findings in a larger sample of nurses working in different hospitals. A replication will possibly reveal differences between hospitals in terms of the organizational job stressors and thus in the nurses' professional commitment. Moreover, the diary entries differed among the nurses. Although controlling for the hierarchical data structure, it might be that the most committed nurses filled out more diaries than less committed nurses. Second, one of the crucial organizational job stressors was negative support of colleagues, which was derived from self‐reported data. Working in clinical practice, however, means that nurses have a position in their team that depends on their personal characteristics and commitment (cf. Brouwer, Flache, Jansen, Hofman, & Steglich, 2018). Therefore, it seems useful to investigate the professional commitment of nurses in their social network to obtain a better understanding of the underlying social mechanisms of professional commitment development and turnover risks.

## 5 CONCLUSION

In summary, in anticipation of growing nursing shortages, it is essential to prevent turnover of novice nurses. Therefore, nurses need a supportive work environment for coping with the most crucial organizational job stressors to enhance professional commitment. In particular, support in the clinical environment is crucial because not feeling supported by colleagues, negative experiences with patients, encountering existential events and conflicting job demands proved to be critical to professional commitment. Retaining novice nurses by creating a supportive work environment for the nursing workforce can be considered a major challenge for nurse managers, organizational management and policymakers.

## CONFLICT OF INTEREST

No conflict of interest has been declared by the authors.

## AUTHOR CONTRIBUTIONS

All authors have agreed on the final version and meet at least one of the following criteria (recommended by the ICMJE [ http://www.icmje.org/recommendations/]): substantial contributions to conception and design, acquisition of data, or analysis and interpretation of data; drafting the article or revising it critically for important intellectual content.

## Supporting information

Appendix
