# Supplementary material for: Turnover prevention: The direct and indirect association between organizational job stressors, negative emotions and professional commitment in novice nurses
Source: J Adv Nurs. 2019 Dec 17;76(3):836–45. doi: 10.1111/jan.14281 (PMC7028052; doi:10.1111/jan.14281)
Supplement: Supplementary file 1 — Appendix [file JAN-76-836-s001.docx]

**Appendix**

Table A1. Summary of unstandardized coefficients and standard errors for the final model.

|  |  |  | 95% confidence interval | |
| --- | --- | --- | --- | --- |
|  | Unstandardized coefficients | Standard error | Lower bound | Upper bound |
| *Professional commitment^1^* |  |  |  |  |
| Negative emotions | -0.08** | 0.02 | -0.12 | -0.04 |
| Conflicting job demands (cognitive) | -0.86** | 0.18 | -1.22 | -0.51 |
| Lack of control | -0.49** | 0.13 | -0.76 | -0.27 |
| *Negative emotions^1^* |  |  |  |  |
| Lack of support from colleagues | 1.48** | 0.18 | 1.13 | 1.81 |
| Negative experience with patients | 1.51** | 0.17 | 1.20 | 1.84 |
| Complexity of care | 0.92* | 0.35 | 0.23 | 1.55 |
| Conflicting job demands (cognitive) | 0.63* | 0.31 | 0.02 | 1.25 |
| Existential events | 1.19** | 0.15 | 0.89 | 1.48 |
| Lack of control | 0.87** | 0.21 | 0.47 | 1.29 |
| Work-life imbalance | 1.01** | 0.27 | 0.48 | 1.54 |
|  |  |  |  |  |
| *Indirect paths on professional commitment through negative emotions* |  |  |  |  |
| Lack of support from colleagues | -0.11* | 0.05 | -0.19 | -0.06 |
| Negative experience with patients | -0.12* | 0.04 | -0.12 | -0.06 |
| Complexity of care | -0.07* | 0.02 | -0.16 | -0.02 |
| Conflicting job demands (cognitive) | -0.05* | 0.02 | -0.13 | -0.01 |
| Existential events | -0.09* | 0.04 | -0.15 | -0.05 |
| Lack of control | -0.07* | 0.02 | -0.13 | -0.03 |
| Work-life imbalance | -0.08* | 0.03 | -0.16 | -0.03 |
|  |  |  |  |  |

*Note. n* = 580; *N* = 18; ***p* ≤ .001; **p* ≤ .05. ^1^Endogeneous variables.
